# A Case of Wernicke’s Encephalopathy Due to Idiopathic Gastroparesis: A Rare Cause of Encephalopathy in a Young Woman

**DOI:** 10.7759/cureus.25653

**Published:** 2022-06-04

**Authors:** Matthew Koury, Julianna Tantum, Corey Savard, Joshua Donohue

**Affiliations:** 1 Internal Medicine, Philadelphia College of Osteopathic Medicine, Philadelphia, USA; 2 Internal Medicine, Lankenau Medical Center, Wynnewood, USA

**Keywords:** vitamin b1, non-alcoholic wernicke’s encephalopathy, thiamine, gastroparesis, wernicke’s encephalopathy

## Abstract

Wernicke’s encephalopathy (WE) is a rare, life-threatening neurological disease due to thiamine deficiency. It is most commonly associated with chronic alcoholism but is also associated with disorders of malabsorption and malnutrition. We present a case of a young female with idiopathic gastroparesis who developed Wernicke’s encephalopathy due to poor oral intake and malnutrition as a result of gastroparesis. This case exemplifies that Wernicke’s encephalopathy should be on the differential in patients who present with encephalopathy with a history of gastroparesis.

## Introduction

Wernicke’s encephalopathy (WE) is a rare neurological disorder associated with the triad of altered mental status, cerebellar dysfunction, and ophthalmologic disorders. WE is caused by thiamine deficiency, which is typically associated with chronic alcohol abuse. However, it has also been associated with disorders of malabsorption and malnutrition, including anorexia nervosa, hyperemesis gravidarum, liver disease, hyperthyroidism, and post-bariatric surgery [1-3]. Cases of gastroparesis leading to WE have rarely been reported [4]. Here we present a case of a young female with a history of idiopathic gastroparesis, who presented with lower extremity weakness, encephalopathy, and nystagmus, who was diagnosed with WE due to her gastroparesis and improved after thiamine supplementation. This case outlines the importance of keeping WE on the differential for patients with a history of gastroparesis who present with encephalopathy.

## Case presentation

This is a 23-year-old African American female with a past medical history of bipolar disorder, on quetiapine, and a recent diagnosis of idiopathic gastroparesis, on cyproheptadine, who presented to the emergency department with progressive bilateral lower extremity weakness for one month, which progressed to ambulatory dysfunction, gait imbalance, and eventually to hypersomnolence two days prior to presentation. She was also experiencing blurry vision and dizziness for the past few months, which were initially attributed to the use of metoclopramide for her gastroparesis diagnosed three months prior to presentation. The patient had a 20-pound weight loss over five months prior to admission due to poor appetite in the setting of poorly controlled gastroparesis. She was also experiencing urinary retention, abdominal bloating, and distention, and her last bowel movement was about one week prior to presentation. She denied a history of trauma, personal or family history of multiple sclerosis, saddle paresthesia, diabetes, bowel or bladder incontinence, neck pain, fevers, chills, recent illness, or sick contacts. She denied any current or previous tobacco use. She denied any current or recent alcohol use but reported drinking alcohol socially one year prior. She received two doses of a COVID-19 vaccine six months prior to presentation.

**Figure 1:**
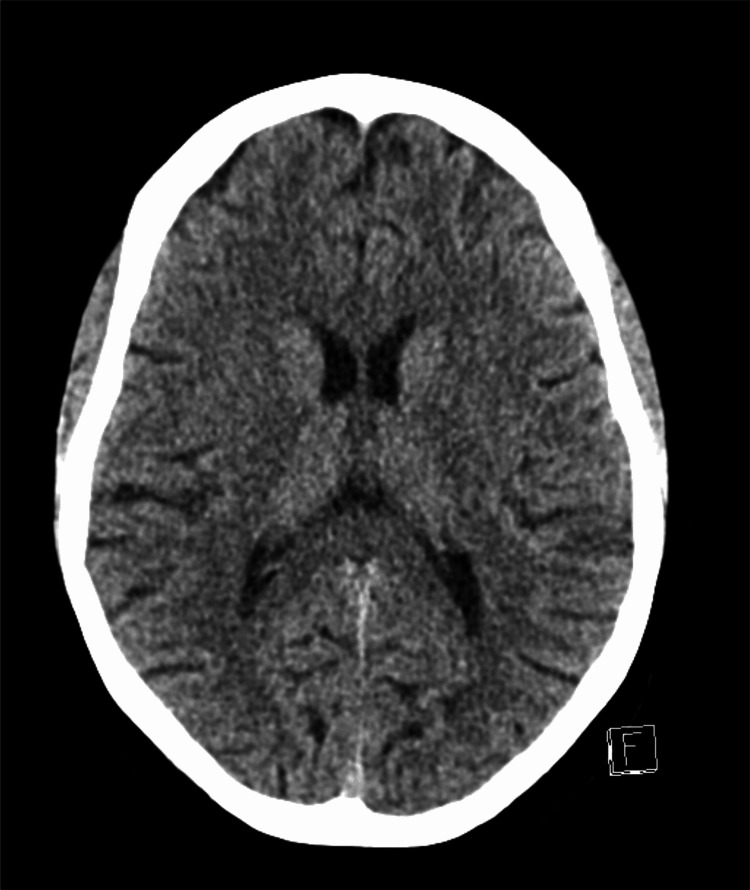
Computerized tomography (CT) scan of the brain in the axial view in a comparable view to the MRIs in Figure 2 and Figure 3, showing no acute abnormality.

On examination, she was hypertensive and tachycardic. She was somnolent, noted to have pinpoint pupils, increased salivation, central obesity, and abdominal and upper limb striae. The abdomen was diffusely tender. Rectal tone was intact. Lower extremity reflexes were absent bilaterally and had a 2/5 strength in her bilateral lower extremities. Notable laboratory values are present in Table 1. Urine drug screen, urinalysis, alcohol, salicylate, acetaminophen levels, and serum pregnancy test were negative. Albumin, total protein, and morning cortisol were normal. Computed tomography (CT) head without contrast showed no acute abnormalities (Figure 1). A plain abdominal radiograph showed no evidence of bowel obstruction. A lumbar magnetic resonance imaging (MRI) with contrast was unremarkable. She was treated with fluids, and a foley was placed for urinary retention. Quetiapine and cyproheptadine were held due to concern for possible medication overdose or anticholinergic toxicity.

**Table 1:** Relevant laboratory values on hospital admission. Patient’s baseline creatinine was 1.0 mg/dL.

Labs	Value	Reference Range
Potassium	3.1 mEQ/L	3.6-5.1 mEQ/L
Bicarbonate	20 mEQ/L	22-32 mEQ/L
Anion Gap	20 mEQ/L	3-15 mEQ/L
Creatinine	1.6 mg/dL	0.6-1.1 mg/dL
Beta-hydroxybutyrate	3.05 mmol/L	<0.28 mmol/L
Glucose	238 mg/dL	70-99 mg/dL
Hemoglobin A1C	5.9%	<5.7%
Lactate	3.8 mmol/L	0.4-2.0 mmol/L
Vitamin B12	629 pg/mL	180-914 pg/mL
Folate	3.1 ng/mL	>=5.8 ng/mL
White Blood Cells	12.3 K/uL	3.8-10.5 K/uL
Thyroid-stimulating Hormone	1.82 mIU/L	0.34-5.6 mIU/L

**Figure 2:**
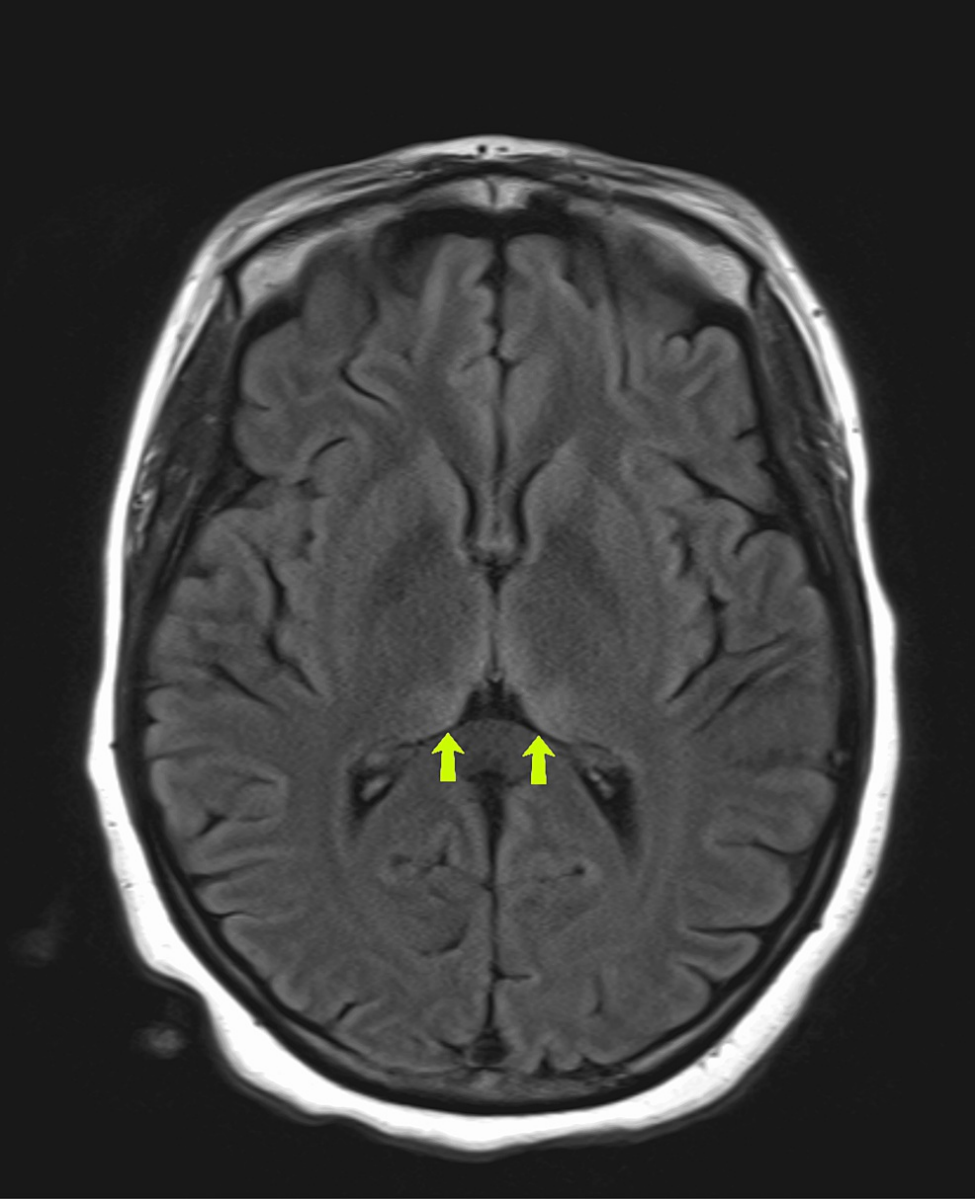
Magnetic resonance imaging (MRI) brain with and without contrast showing increased T2 signal in the medial thalamus bilaterally (yellow arrows), consistent with Wernicke’s encephalopathy performed during hospital admission.

**Figure 3:**
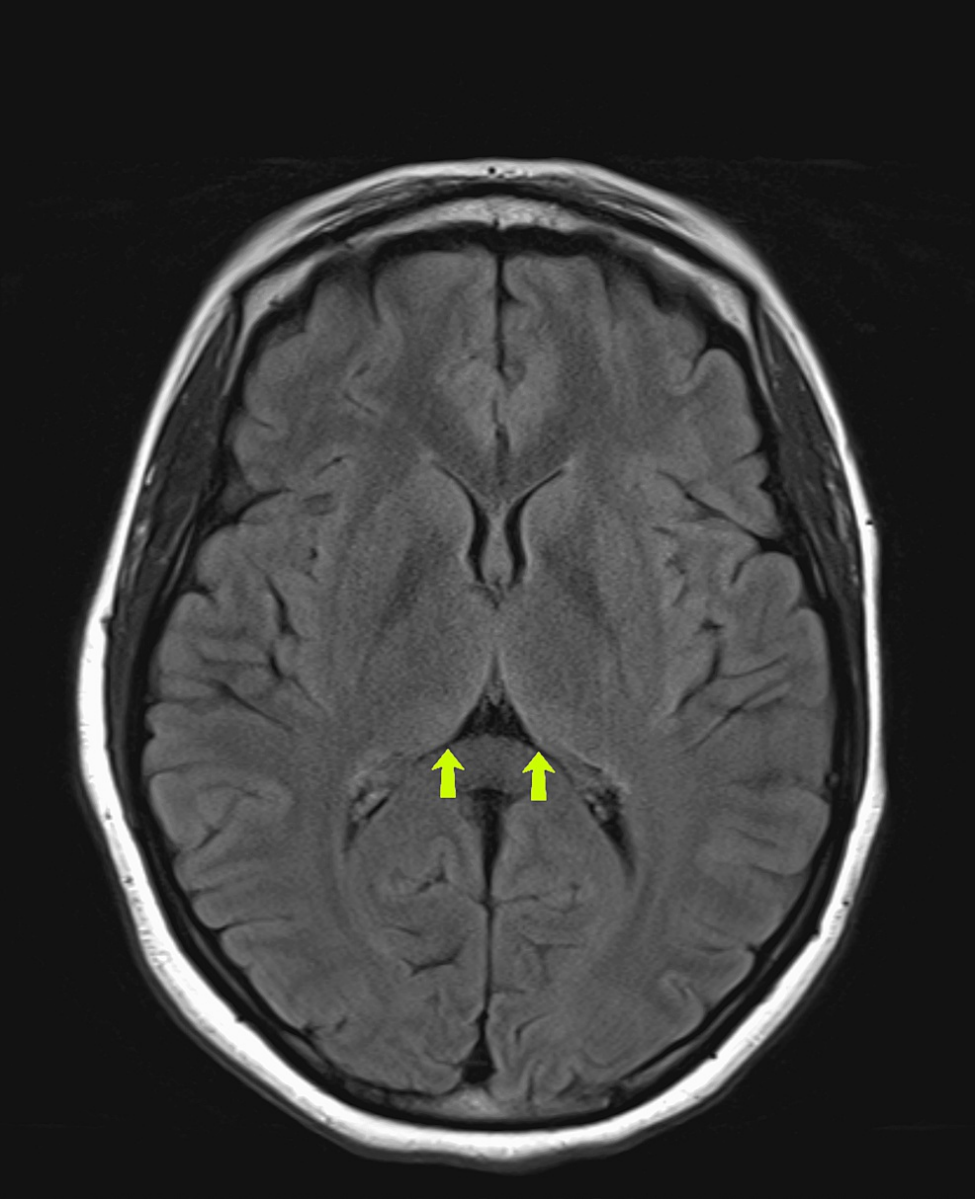
Magnetic resonance imaging (MRI) brain with and without contrast showing improving T2 signal in the medial thalamus bilaterally with mild residual abnormal increased T2 signal persisting (yellow arrows), performed approximately seven months after hospital admission.

On hospital day 1, her encephalopathy continued, and she underwent a lumbar puncture with no evidence of infection. Human immunodeficiency virus (HIV) and rapid plasma reagin (RPR) testing were negative. On hospital day 2, the patient gradually became more alert and was found to have severe sustained conjugate vertical nystagmus, superimposed on severe disconjugate horizontal gaze with severe end gaze nystagmus. MRI of the brain with and without contrast showed increased T2 signal with restricted diffusion in the medial thalami bilaterally, surrounding the third ventricle, and in the periaqueductal gray matter, consistent with WE (Figure 2). She was started on high-dose thiamine, folate, and multivitamin. With treatment, her encephalopathy, nystagmus, and weakness improved. She was discharged to a rehabilitation facility with lifelong vitamin supplementation and off all psychotropic medications until psychiatry follow-up, given the concern for malabsorption due to her gastroparesis. Seven months from the initial presentation, the patient continues to recover with increasing mobility, resolving nystagmus, and an improving MRI (Figure 3).

## Discussion

WE is a rare neurological condition with a high degree of morbidity and mortality. The typical manifestations of WE include the triad of altered mental status, cerebellar dysfunction, and ophthalmologic disorders, typically manifesting as nystagmus. However, only 16% of patients with WE have the classic triad of symptoms, making diagnosis challenging, especially in non-alcoholics [1,4,5]. WE is caused by thiamine deficiency. Thiamine, also known as Vitamin B1, is an essential cofactor involved in the Krebs cycle and pentose phosphate pathway. Prolonged thiamine deficiency can lead to oxidative stress and neuronal necrosis, eventually leading to the symptoms of WE [6]. WE and thiamine deficiency are most commonly associated with chronic alcoholism but have also been associated with malnutrition and malabsorption. Cases of WE have been reported in patients with anorexia nervosa, hyperemesis gravidarum, gastric outlet obstruction, and post-bariatric surgery [1,3,7]. Few cases have reported an association with WE and gastroparesis [4].

Given the patient’s history, clinical findings, and combination of medications (quetiapine, cyproheptadine, and previously metoclopramide), anticholinergic toxicity or a medication overdose was initially suspected. However, many of her symptoms did not align with this diagnosis. After vertical nystagmus was appreciated and brain MRI was obtained, the diagnosis of WE was made. With high-dose thiamine, her symptoms of encephalopathy, weakness, and nystagmus gradually improved. It was suspected that her poorly controlled idiopathic gastroparesis led to poor oral intake over several months with subsequent weight loss, resulting in severe malnutrition and Vitamin B1 deficiency. Of note, the cause of the patient’s gastroparesis has remained unclear, but the commonly known associations with gastroparesis, alcohol abuse, tobacco, and diabetes, have been ruled out in her [8].

There have been few reported cases of gastroparesis leading to WE, especially in patients so young and with such an acute presentation [5]. Our patient presented with several of the most common symptoms of WE: confusion, oculomotor dysfunction, polyneuropathy, and nystagmus. She also presented with the non-specific findings of vomiting and severe weight loss central to the presentation of nonalcoholic WE [9]. However, the suspected medication overdose confounded the initial diagnosis. In nonalcoholic patients, WE can be overlooked as a diagnosis, especially in such a young patient [3,5].

## Conclusions

This unique case demonstrates that patients with gastroparesis can develop severe nutritional deficiencies resulting in WE. It remains unclear whether lack of intake or malabsorption of thiamine led to her WE, but in either case, it was a result of her gastroparesis. Given the high degree of morbidity and mortality associated with WE, this diagnosis should remain on the differential in patients with a neurological presentation in the setting of a gastroenterological abnormality.
